# Generation of Functional Myocytes from Equine Induced Pluripotent Stem Cells

**DOI:** 10.1089/cell.2018.0023

**Published:** 2018-09-27

**Authors:** Karin R. Amilon, Yennifer Cortes-Araya, Benjamin Moore, Seungmee Lee, Simon Lillico, Amandine Breton, Cristina L. Esteves, F. Xavier Donadeu

**Affiliations:** ^1^ The Roslin Institute and R(D)SVS, University of Edinburgh, Edinburgh, United Kingdom.; ^2^ The Euan Macdonald Centre for Motor Neurone Disease Research, University of Edinburgh, Edinburgh, United Kingdom.

**Keywords:** iPSC, equine, veterinary, myocyte, myotube, MYH, skeletal muscle

## Abstract

Induced pluripotent stem cells (iPSCs) have revolutionized human biomedicine through their use in disease modeling and therapy. In comparison, little progress has been made toward the application of iPSCs in veterinary species. In that regard, skeletal myocytes from iPSCs would have great potential for understanding muscle function and disease in the equine athlete. In this study, we generated skeletal myotubes by transducing equine iPSC-derived mesenchymal derivatives with an inducible lentiviral vector coding for the human sequence of the myogenic factor, MyoD. Myosin heavy chain-positive myotubes generated from two different iPSC lines were compared to myotubes from adult equine skeletal muscle progenitor cells (MPCs). iPSC myotubes had a smaller mean area than MPC myotubes (≤2-fold). In addition, quantitative polymerase chain reaction analyses showed that iPSC myotubes expressed *MYH2* and *MYH3* isoforms (at similar or lower levels than MPC myotubes), but they did not express the mature muscle isoform, *MYH1*. Compared to MPC myotubes, iPSC myotubes expressed reduced levels of the myogenic factors, *MYOD1* and *MYF6*, but did not express *MYF5*. Finally, iPSC myotubes responded to KCl-induced membrane depolarization by releasing calcium and did so in a manner similar to MPC myotubes. In conclusion, this is the first study to report the generation of functional myocytes from equine iPSCs.

## Introduction

Despite the importance of healthy skeletal muscle for the equine athlete, little is known about the mechanisms underpinning its development and disease mechanisms. A particularly important issue for the horse industry is the relatively high incidence of equine-inherited myopathies, including polysaccharide storage myopathy (PSSM1), hyperkalemic periodic paralysis (HYPP), and recurrent exertional rhabdomyolysis, a consequence of historic selection for desired performance traits in some horse breeds such as the Quarter Horse (Mickelson and Valberg, 2015). Some of those myopathies also occur in other species, including humans.

Given the difficulties associated with performing studies *in vivo*, progress toward understanding their pathogenesis would be significantly facilitated by the availability of robust *in vitro* disease models allowing functional testing of already identified candidate gene mutations, for example, in the case of PSSM1 and HYPP. Significant effort has already been put into developing such models using primary (Baquero-Perez et al., 2012) or immortalized (Naylor and Piercy, 2015) muscle cells or muscle-like cells generated through forced transdifferentiation of skin cells (Fernandez-Fuente et al., 2008). However, the applicability of these models to study disease is limited because they require biopsy sampling of patient tissues, which is not always possible, and the cells have limited lifespan and/or a restricted ability to replicate the native myocyte phenotype *in vitro*, thus precluding meaningful gene function analyses.

In that regard, given their ability to proliferate indefinitely *in vitro*, high developmental plasticity and amenability to robust genetic manipulation, using novel gene editing technologies, induced pluripotent stem cells (iPSCs) offer a unique tool for understanding the effects of disease-causing genetic mutations and for testing novel therapeutic targets through what has been called “disease in a dish” (Hockemeyer and Jaenisch, 2016).

iPSCs are already being used for modeling human skeletal muscle disease, for example, Duchenne muscular dystrophy and type 2 diabetes (Choi et al., 2016; Iovino et al., 2016), and functional myogenic precursors have been generated that can efficiently engraft and promote muscle regeneration in animal models (Darabi et al., 2012). Functional muscle cells have been generated from human or rodent iPSCs/embryonic stem cells (ESC) by forced expression of ectopic myogenic genes, primarily Pax7 or MyoD (Chal and Pourquie, 2017), or, more recently, using chemical approaches that aim to replicate muscle development in the embryo to produce genetically unmodified cells that could be ideally used for developmental muscle studies (Chal et al., 2016; Xu et al., 2013).

Compared to humans, the availability of iPSCs (and specially ESCs) from horses is limited, as is the number of studies reporting their differentiation into functional cell types (Donadeu, 2014). A previous study showed the generation of myosin heavy chain (MyHC)-positive myotubes from equine skeletal muscle cell-derived iPSCs (Quattrocelli et al., 2016), whereas another reported formation of muscle fibers after transplantation of equine adipose stem cell-derived iPSCs into injured skeletal muscle of mice (Lee et al., 2016). In the present study, we took a step further by generating functional myocytes from equine iPSCs and comparing their characteristics with those of myocytes produced from adult equine skeletal muscle precursors.

## Materials and Methods

### Cell derivation and culture

Two different iPSC lines (H and U) derived from equine fibroblasts in our laboratory (Breton et al., 2013) were used in this study. iPSCs were maintained on Matrigel™ (BD Biosciences) in conditioned medium generated from inactivated equine fetal fibroblasts (Sharma et al., 2014) comprising knockout-DMEM (Dulbecco's modified Eagle's medium) (Gibco), 20% knockout serum replacement (Gibco), 0.1 mM β-mercaptoethanol, 0.1 mM nonessential amino acids (Gibco), 2 mM L-glutamine (Gibco), 1000 U/mL leukemia inhibitory factor (LIF; Sigma-Aldrich), and 8 ng/mL human basic fibroblast growth factor (bFGF; Sigma-Aldrich). Cells were in all cases cultured at 37°C in a humidified atmosphere with 5% CO_2_ and unless specified, medium was replaced every 1–2 days.

Skeletal muscle was obtained postmortem from an adult horse euthanized for unrelated reasons at the Royal (Dick) School of Veterinary Studies, University of Edinburgh. The tissue was cut into small pieces (∼3 cm^2^), washed in phosphate-buffered saline (PBS), and minced using sterile forceps and scissors before digestion with 1% Protease in high-glucose DMEM (Sigma-Aldrich) at 37°C for 1 hour, with shaking at 100 rpm.

Digested tissue was then centrifuged at 400 *g* for 5 minutes, and the resulting tissue/cell pellet was suspended in high-glucose DMEM containing 10% fetal bovine serum (FBS; Gibco) and 1% penicillin–streptomycin (PS; Gibco), and passed vigorously through a 10 mL pipette about 20 times before allowed to settle. The supernatant was transferred to a fresh tube, and the remaining pellet was passed repeatedly through a 5 mL pipette before another supernatant was collected. The pooled supernatants were filtered through a 40 μm cell strainer and subsequently centrifuged at 1000 *g* for 10 minutes, after which the resulting cell pellet was plated onto 0.2% gelatin (Sigma-Aldrich) in high-glucose DMEM containing 10% FBS and 1% PS.

Cells were cultured for three to four passages before induced to differentiate using an adaptation of the protocol by Chen et al. (2015). In brief, cells (2 × 10^3^/cm^2^) were seeded on collagen (Sigma-Aldrich) in skeletal proliferation medium containing high-glucose DMEM, 10% FBS, 10% horse serum (HS; Gibco), 1% chicken embryo extract (CEE, CE-650-J; Seralab), and 1% PS. When 80% confluent, cells were trypsinized, counted, and again seeded at the same density on collagen and cultured in the same media until 80% confluent, after which cells were lifted and seeded at 2 × 10^4^ cells/cm^2^ on collagen in skeletal differentiation medium containing high glucose-DMEM, 1% FBS, 1% HS, 0.1% CEE, and 1% PS. Cells were differentiated for 7 days before samples were collected for immunocytochemistry and mRNA analysis.

### Chemically induced iPSC differentiation

Equine iPSCs were differentiated as described for human iPSCs by Chal et al. (2016). In brief, cells were seeded in Matrigel-coated 12-well plates (70,000 cells/well) in mTeSR1 media (Stem Cell Technologies [STC]) on day 1, after which they were incubated in DMEM containing ITS (1/100; Gibco), GSK-inhibitor (CHIRON, 3 μM; STC), and ALK inhibitor (LDN-193189, 0.5 μM; Stemgent) between days 2 and 5, with bFGF (20 ng/mL) added for the last 2 days.

Cells were then changed to DMEM containing HGF (10 ng/mL; Biolegend), IGF1 (2 ng/mL; Sigma-Aldrich), bFGF, and LDN-193189 until day 7 and then placed in DMEM with knockout serum replacement and IGF-1 to which bFGF was added on day 12, under which cells were maintained up to at least day 30. Samples were collected for immunochemistry and quantitative polymerase chain reaction (qPCR) analyses as described below.

### iPSC differentiation using MyoD lentivirus

Lentiviral particles were generated by cotransfecting HEK293T cells with LV-TRE-WT human MyoD-T2A-dsRedExpress2 obtained from Addgene (plasmid 60628) (Kabadi et al., 2015), the packaging plasmid psPAX2, and VSVG-plasmid using FuGENE^®^ HD Transfection Reagent (Promega). The MyoD-coding plasmid contains a puromycin resistance gene sequence. Cells were incubated for 48 hours, after which the supernatant was harvested, passed through a 0.45 μm filter and concentrated by ultracentrifugation. The final virus suspension was stored as single use aliquots at −80°C. The viral titer was determined by transducing HT1080 cells with serial dilutions of the viral stock solution for 24 hours, followed by Puromycin (1 μg/mL; Sigma-Aldrich) selection and counting of positive transductants. Viral transducing units were calculated to be 1.7 × 10^6^/mL.

Equine iPSCs were trypsinized and seeded at 15,000 cells/cm^2^ in iPSC conditions (see above) before they were transduced as described below. Alternatively, before transduction, iPSCs were trypsinized and seeded onto 0.2% gelatin in high-glucose DMEM containing 10% FBS and 1% PS to allow spontaneous differentiation, and 14 days later they were trypsinized and seeded at a density of 5000 cells/cm^2^ and allowed to adhere overnight.

In all cases, cells were then transduced with 5 μg/mL polybrene (Santa Cruz Biotechnology) and lentivirus at multiplicities of infection between 2 and 6 for 24 hours, after which the cells were washed with PBS and refreshed with culture medium containing 1–2 μg/mL puromycin for selection and expansion of positive transductants. Before transduction, a kill curve was performed to determine optimal Puromycin concentration for each cell type.

Puromycin-resistant cells were seeded in 12-well plates (40,000/well) in DMEM high glucose containing 10% FBS and 1% PS. The following day, doxycycline (Sigma-Aldrich) was added to a concentration of 3 μg/mL, and cells were differentiated for 7 days as described above for skeletal muscle cells, after which samples were collected for immunocytochemistry, qPCR, or calcium analyses.

### Immunocytochemistry

Cells were fixed and permeabilized in ice-cold methanol:acetone (50:50) solution for 10 minutes at room temperature, followed by washing with PBS for 3 × 5 minutes and incubated with protein block solution (Springbio) for 1 hour at room temperature. Cells were stained with anti-MyHC antibody (10 μg/mL, #MF20 MAB4470; R&D Systems) in antibody diluent reagent (Invitrogen) at 4°C overnight. Cells were washed with PBS to remove any unbound antibody and incubated with AF488-conjugated goat anti-mouse IgG (A11029; Invitrogen) for 1 hour at room temperature and kept in the dark. Cells were washed as before and mounted in Fluoroshield with DAPI (Sigma-Aldrich), sealed with a coverslip and examined using a Zeiss Axiovert 25 inverted fluorescent microscope.

Pictures were taken using a Zeiss Axiocam 503 high-resolution color camera/Zen software. Myotube area and number of myonuclei were determined from MYH-stained pictures using ImageJ software; for each cell type, mean values were taken from four myotubes analyzed from each of four pictures.

### Quantitative polymerase chain reaction

Cells were harvested into TRIzol (Thermo Fisher Scientific), and RNA was extracted according to the manufacturer's protocol. Total RNA was quantified by Nanodrop (Thermo Scientific), and 1 μg was reverse transcribed using Superscript III (Thermo Fisher scientific). qPCR was performed using SensiFAST SYBR Lo-ROX Kit (Bioline) in a MX3005P system (Stratagene) and data analyzed with MxPro Software. Expression for each gene was determined using standard curves prepared from skeletal muscle or pooled samples and normalization to the expression of *18S* within each sample. Primers used are listed in Table 1.

**Table 1.** List of Primers Used for Quantitative Polymerase Chain Reaction Analyses

*Gene*	*Forward primer (5′-3′)*	*Reverse primer (5′-3′)*
18S	GCTGGCACCAGACTTG	GGGGAATCAGGGTTCG
*MYH1*	CACTTCAAGGCCGCATCTCTA	AACTCATGGCTGCGGGTTAT
*MYH2*	GGAGGCTGAGGAACAATCCA	CTGTGCCTCTCTTCAGTCATTC
*MYH3*	CCGAGGAGGCTGATGAACAA	CGCTCACTCTTCGCTCTCAT
*MYOD1*	GCAAGCGCAAGACCACTAAC	GGCTTCGTTGACTTTGCTCA
*MYF5*	TGTTCAGAGCCCACTAGCC	GGTGATCCGATCCACTATGC
*MYF6*	CTCGTGATAACCGCCAAGGA	CGATGGAAGAAAGGCATCGA
Human *MYOD1*	CCGACGGCATGATGGACTAC	AGGCAGTCTAGGCTCGACAC

### Calcium assay

Fluo-4 Direct™ Calcium Assay Kit (Invitrogen) was used according to the manufacturer's protocol to fluorescently label myotubes to monitor Ca^2+^ release in response to membrane depolarization with 75 mM KCl. Myotubes were examined under a Zeiss Axiovert 25 inverted fluorescent microscope and pictures were taken before and 1 minute after addition of KCl using a Zeiss Axiocam 503 high-resolution color camera with Zen software. Average Fluo-4 intensity was calculated from images from five to six myotubes analyzed using ImageJ.

### Statistical analyses

Data normality was assessed by Kolmogorov–Smirnoff test, and data were log-transformed before analyses if needed. Data were then analyzed using the GLM (generalized linear model) procedure by one-way ANOVA followed by Tukey's pairwise comparison tests. In all cases, statistical significance was considered at *p* < 0.05.

## Results and Discussion

### Generation of myotubes from iPSCs

We first tested a directed differentiation approach successfully used to generate functional skeletal muscle from mouse and human PSCs and that recapitulates paraxial mesoderm specification and differentiation by manipulating key signaling pathways such as WNT and BMP (Chal et al., 2016). Although cells adopted an elongated morphology under these conditions, very few multinucleated structures were observed during the 50-day differentiation protocol, and no expression of progenitor (*MYOD1*, *MYF5*) or differentiated (*MYH1*, *MYH2*, *MYH3*) muscle cell transcripts was detected by qPCR in these cultures, indicating that these conditions are not optimum for promoting myogenic differentiation of equine iPSCs.

We then decided to use a transgenic approach involving expression of inducible MyoD. To that end, we transduced iPSCs with LV-TRE-WT human MyoD-T2A-dsRedExpress2. Although some cells exhibited red fluorescence after addition of doxycycline, there were no signs of myogenesis after puromycin-selected cells were cultured in differentiation media. This could be partly attributed to low efficiency of iPSC transfection, as indicated by the presence of only a few red fluorescence cells. Moreover, previous studies in other species (Albini et al., 2013; Goudenege et al., 2012) have shown the requirement for pluripotent cells to transition to a mesodermal stage before they become epigenetically competent to initiate myogenesis in response to MyoD (Albini et al., 2013).

**FIG. 1.**
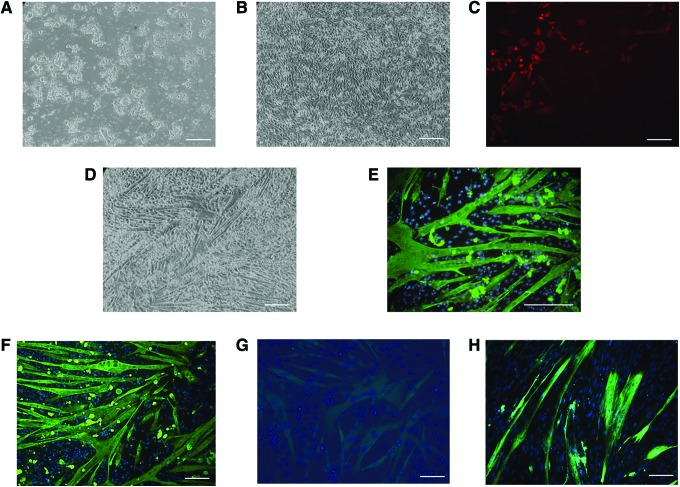
Representative micrographs of equine iPSCs before **(A)** and after **(B)** differentiation for 14 days in 10% FBS, at which point, cells were transduced with the LV-TRE-WT human MyoD-T2A-dsRedExpress2 construct followed by puromycin selection. Treatment of selected cells with doxycycline resulted in the appearance of red fluorescent cells within 1 day **(C)** and the formation of abundant myotubes **(D)** that stained positive for MyHC **(E)**. Myotubes generated from iPSC lines H **(D–F)** and U **(G**, which displayed only mild MyHC staining) were compared to those generated from adult MPCs **(H)**. *Green* = MyHC, *blue* = DAPI. Scale bar: 50 μm. iPSC, induced pluripotent stem cell; MyHC, myosin heavy chain; MPCs, muscle progenitor cells. Color images available online at www.liebertpub.com/cell

Therefore, we decided to introduce a differentiation step to generate mesenchymal-like cells, which we reasoned would be both easier to transduce and already primed for myogenesis. To achieve this, iPSCs (Fig. 1A) were placed in DMEM-containing 10% FBS for 14 days in the absence of LIF before transduction with LV-TRE-WT human MyoD-T2A-dsRedExpress2 (Fig. 1B). One day after adding doxycycline, we could observe red-fluorescent cells (Fig. 1C) that followed a few days later by formation of myotubes that stained for MyHC (Fig. 1D–G). We used this strategy with two different iPSC lines, H and U, although with variable efficiency and were able to maintain these myotubes in culture without detaching or other signs of cell death for at least 7 days, even after doxycycline had been removed.

### Characteristics of myotubes derived from equine iPSCs

**FIG. 2.**
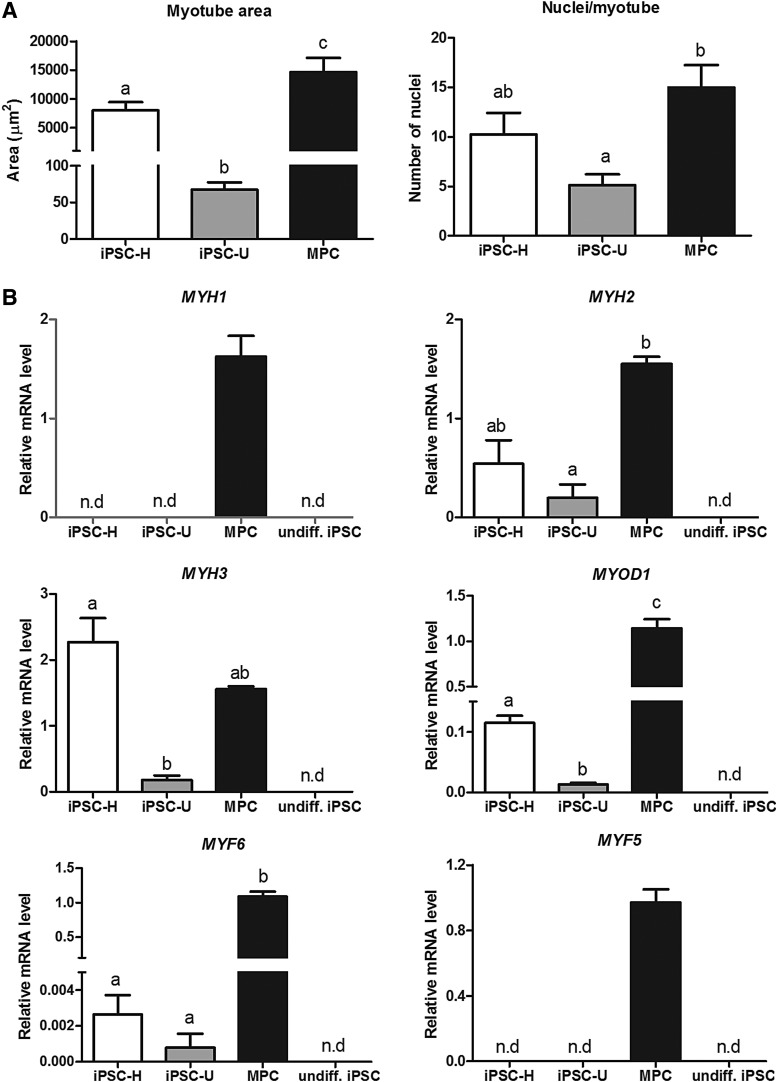
Characteristics of myotube cultures generated from equine iPSC lines H and U, and from adult MPCs in relationship to **(A)** myotube area and number of myonuclei (*n* = 16 myotubes per cell type) and **(B)** transcript levels (normalized to 18S) of MyHC isoforms (*MYH1*, *MYH2*, and *MYH3*) and myogenic regulatory factors (*MYOD*, *MYF6*, and *MYF5*, *n* = 3 independent cultures per cell type). Undiff iPSCs, undifferentiated parental iPSCs. Values are shown as mean ± SE. Means with *different superscripts* (a–c) are different (*p* < 0.05). n.d, not detected; SE, standard error.

To characterize myotubes obtained from iPSC-derived mesenchymal cells (hereafter referred to as iPSC myotubes), we compared their properties with those of myotubes generated spontaneously from progenitor cell cultures obtained from adult equine muscle (muscle progenitor cells [MPC] myotubes, Fig. 1H). iPSC myotubes were smaller than MPC myotubes, as indicated by smaller mean areas, particularly in the case of U-line myotubes, and smaller mean numbers of myonuclei per myotube (only statistically significant for U-line myotubes, Fig. 2A).

To investigate the developmental stage of iPSC compared with MPC myotubes, we performed qPCR analyses of selected genes (Fig. 2B), including embryonic (*MYH3*) and adult (*MYH1*, *MYH2*) MyHC isoforms, as well as *MYF5*, *MYOD1*, and *MYF6* (also known as *MRF4*), three myogenic regulatory factors (MRFs) that are temporally expressed in this order during fetal myogenesis (Bentzinger et al., 2012). Compared with MPC myotubes, iPSC myotubes expressed *MYH3* and *MYH2*, but not *MYH1*, an isoform that is naturally expressed at a relatively late stage (late fetal or postnatal) during normal muscle development (Schiaffino et al., 2015). Expression of *MYH2* and *MYH3* by iPSC line H myotubes was similar (*p* > 0.1) to that by MPC myotubes, but was comparatively lower (*p* < 0.05) in iPSC line U myotubes.

Moreover, consistent with their reduced size and lack of expression of mature MYH isoform, the expression of *MYOD1* and *MYF6* by iPSC myotubes was in general much lower than by MPC myotubes, suggesting only partial activation of the myogenic transcriptional program in response to ectopic MyoD expression in iPSC-derived cells. *MYF5* was not detected in myotubes from any of the two iPSC lines. This is the first MRF to be expressed during muscle development in the embryo, although it has been shown to be functionally redundant with the downstream gene, MyoD, so that in the absence of Myf5, myogenesis can proceed normally once MyoD has been activated (Bentzinger et al., 2012). Consistent with this, activation of endogenous Myf5 was not required for myogenesis to occur in response to ectopic MyoD in our study.

Overall, the reduced myogenic response from U- compared with H-line iPSCs may reflect intrinsic differences in their epigenetic landscape imposed during reprogramming, and may also have been resulted, at least in part, from mean lower relative expression (although not statistically significant) of the virus-driven human MyoD transgene in U compared with H cells (3.6-fold lower, *p* = 0.06, *n* = 3 experiments).

**FIG. 3.**
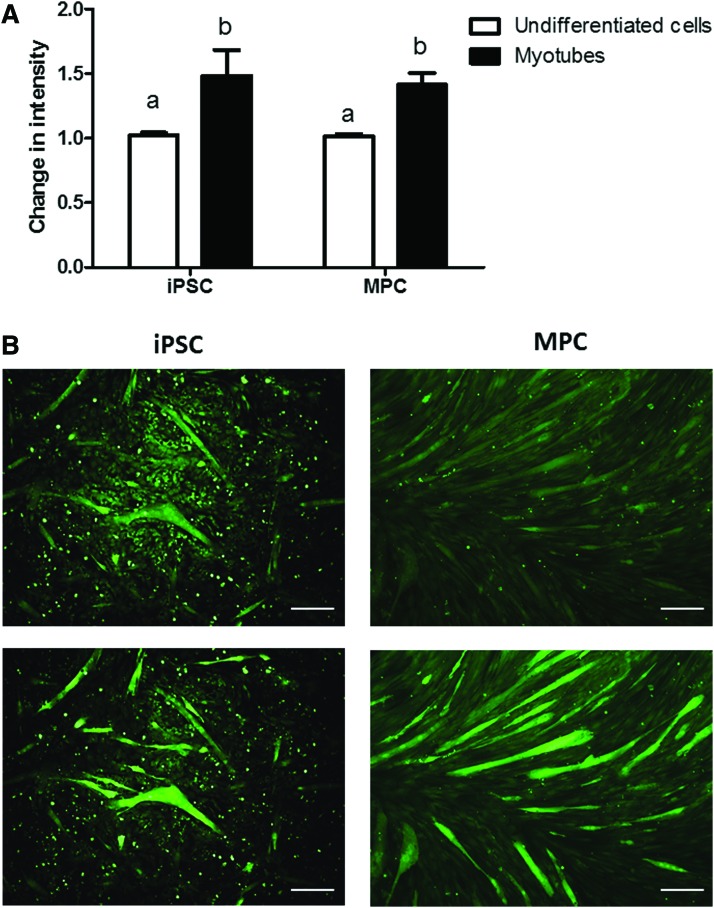
**(A)** Calcium responses (expressed as mean ± SE change in Fluo-4 intensity) to depolarization with 75 mM KCl of myotubes derived from iPSCs (line H) or adult MPCs (both shown as *black bars*) and undifferentiated iPS-derived cells or MPCs (shown by *white bars*, *n* = 5–6 myotubes per cell type). Means with *different superscripts* (a, b) are different (*p* < 0.05). **(B)** Representative micrographs showing changes in fluorescence before (*upper panels*) and 1 minute after (*lower panels*) addition of 75 mM KCl to iPSC- or MPC myotube cultures. Scale bar: 50 μm. Color images available online at www.liebertpub.com/cell

Finally, we assessed the electrophysiological properties of the myotubes generated *in vitro* by measuring intracellular calcium release in response to KCl-induced membrane depolarization, which is a measure of functional coupling between plasma membrane voltage-dependent calcium channels and calcium release from the sarcoplasmic reticulum membrane of skeletal muscle cells. A calcium response was elicited by membrane depolarization of iPSC myotubes as it was from MPC myotubes (Fig. 3), indicating a degree of electrophysiological maturation in iPSC myotubes and in agreement with previous data with human iPSCs (Skoglund et al., 2014).

In conclusion, this is the first report of the generation of functional muscle cells from equine iPSCs, as indicated by the ability of myotubes to respond to a membrane depolarization stimuli. Compared with myotubes generated from adult MPCs, iPSC-derived myotubes displayed an immature phenotype and could thus be particularly useful for developmental and disease pathogenesis studies. Further work should be aimed at optimizing protocols for induction of myogenesis from iPSCs of equine and other large animal species using gene-free strategies, an approach that may lead to the generation of fully mature myotubes *in vitro*.
